# Towards pervasive computing in health care – A literature review

**DOI:** 10.1186/1472-6947-8-26

**Published:** 2008-06-19

**Authors:** Carsten Orwat, Andreas Graefe, Timm Faulwasser

**Affiliations:** 1 Institut für Technikfolgenabschätzung und Systemanalyse (Institute for Technology Assessment and Systems Analysis), Forschungszentrum Karlsruhe in der Helmholtz-Gemeinschaft (Karlsruhe Research Centre, Member of the Helmholtz Association); Address: P.O. Box 3640, D-76021 Karlsruhe, Germany; 2 Institut für Automatisierungstechnik (Institute for Automatic Control), Otto-von-Guericke Universität Magdeburg (Otto-von-Guericke University Magdeburg); Address: P.O. Box 4120, D-39016 Magdeburg, Germany

## Abstract

**Background:**

The evolving concepts of pervasive computing, ubiquitous computing and ambient intelligence are increasingly influencing health care and medicine. Summarizing published research, this literature review provides an overview of recent developments and implementations of pervasive computing systems in health care. It also highlights some of the experiences reported in deployment processes.

**Methods:**

There is no clear definition of pervasive computing in the current literature. Thus specific inclusion criteria for selecting articles about relevant systems were developed. Searches were conducted in four scientific databases alongside manual journal searches for the period of 2002 to 2006. Articles included present prototypes, case studies and pilot studies, clinical trials and systems that are already in routine use.

**Results:**

The searches identified 69 articles describing 67 different systems. In a quantitative analysis, these systems were categorized into project status, health care settings, user groups, improvement aims, and systems features (i.e., component types, data gathering, data transmission, systems functions). The focus is on the types of systems implemented, their frequency of occurrence and their characteristics. Qualitative analyses were performed of deployment issues, such as organizational and personnel issues, privacy and security issues, and financial issues. This paper provides a comprehensive access to the literature of the emerging field by addressing specific topics of application settings, systems features, and deployment experiences.

**Conclusion:**

Both an overview and an analysis of the literature on a broad and heterogeneous range of systems are provided. Most systems are described in their prototype stages. Deployment issues, such as implications on organization or personnel, privacy concerns, or financial issues are mentioned rarely, though their solution is regarded as decisive in transferring promising systems to a stage of regular operation. There is a need for further research on the deployment of pervasive computing systems, including clinical studies, economic and social analyses, user studies, etc.

## Background

### Pervasive computing and related concepts

Pervasive computing, ubiquitous computing, and ambient intelligence are concepts evolving in a plethora of applications in health care. In the literature, pervasive computing is loosely associated with the further spreading of miniaturized mobile or embedded information and communication technologies (ICT) with some degree of 'intelligence', network connectivity and advanced user interfaces [1-5]. Because of its ubiquitous and unobtrusive analytical, diagnostic, supportive, information and documentary functions, pervasive computing is predicted to improve traditional health care [6,7]. Some of its capabilities, such as remote, automated patient monitoring and diagnosis, may make pervasive computing a tool advancing the shift towards home care, and may enhance patient self-care and independent living. Automatic documentation of activities, process control or the right information in specific work situations as supplied by pervasive computing are expected to increase the effectiveness as well as efficiency of health care providers. For example, in hospitals pervasive computing has the potential to support the working conditions of hospital personnel, e.g., highly mobile and cooperative work, use of heterogeneous devices, or frequent alternation between concurrent activities [8]. 'Anywhere and anytime' are becoming keywords – a development often associated with 'pervasive healthcare' [9,10]. On the other hand, the social, economic and ethical concerns regarding the use of pervasive computing may detract from its acceptance and societal desirability, which is equally relevant to health care [11,12].

### Purpose of this review

Pervasive computing entered health care in almost every setting, making it difficult to develop an idea of its typical implementation and maintain an overview of recent developments. We address this difficulty by providing a systematic overview and analysis of systems developments and implementations of pervasive computing in health care and highlighting experiences in deployment. Summarizing published research, this literature review provides a resource for researchers, scholars, or practitioners dealing with pervasive computing. That said, many systems developments and implementations are not published in the literature. Therefore, this article does not fully cover the field of pervasive computing in health care. Rather, it provides an overview of peer-reviewed literature on this topic.

## Methods

### Scope of systems

As the technology is still evolving, there is neither an appropriate definition of pervasive computing [13] nor an exact distinction from similar terms, such as ubiquitous computing [14] or ambient intelligence [15]. This often leads systems developers, imprecisely, to declare their systems 'pervasive' or simply not use any of these terms. Therefore, for this literature review a set of criteria which defined the framework for the selection process had to be developed. The criteria are minimum features of pervasive computing regarded as new and distinctive. This selection seeks to identify articles in which systems with inherent pervasive computing features are covered in line with the criteria outlined below. The selection criteria were defined to include a broad range of different systems. They were also designed so as to distinguish our search and position it in broader concepts, such as telemedicine or e-health. Articles not clearly telling whether the systems described meet the inclusion criteria were not considered.

#### Inclusion criteria for systems

First, systems were included, when they were ubiquitous in the sense of being not bound to one dedicated location, such as a computer at a workplace. For example, systems of telemedicine via video conferencing at dedicated places were not considered (e.g., stationary desktop computers). Instead, systems were included which featured:

• mobile devices (e.g., laptops, PDAs, tablet PCs, mobile phones),

• wearable items (computer-enhanced textiles, accessories, or medical devices),

• implanted devices, as well as

• stationary devices, such as sensors or other ICT components embedded in 'everyday objects' or infrastructure, such as buildings, furniture, etc.

Secondly, systems were considered which had elements of 'intelligence' in the sense of context awareness [16,17] or decision support capabilities. Systems transferring information only by simply forwarding data were excluded, such as PDAs sending manually entered data to a server. Thirdly, data processing or transmission must be performed by systems without any human intervention. For instance, systems requiring manual data conversion, such as printing and manual re-entering of data in the process chain, were excluded.

#### Inclusion criteria for studies

Prototypes, tests, pilot studies and case studies conducted in health care settings, or systems involving prospective end users, clinical trials as well as systems already in routine use were included. Experiments in non-medical settings as well as mere descriptions of concepts, designs or architectures were not included. Only complete functioning systems, no components or parts, were taken into account.

### Search method

This literature review is limited to published work that has undergone scientific peer-review processes. Our search was restricted to articles in journals and chapters of periodicals written in English and published between 2002 and 2006. Keyword searches were conducted in PubMed, ISI Web of Science (Science Citation Index Expanded), IEEE Xplore and INSPEC by using the search string ("pervasive computing" OR "ubiquitous computing" OR "ambient intelligence" OR "pervasive healthcare") AND (healthcare OR "health care" OR medic*). These databases contain, among others things, literature in the fields of medicine, medical informatics, medical technology, computer science and research, as well as electronic engineering. The database searches led to 247 distinct articles. As many authors do not use the terms 'pervasive computing', 'ubiquitous computing', etc., 46 periodicals were searched manually (see Additional file 1). The journals were selected to represent the fields of medical informatics and pervasive computing most relevant to the subject at hand.

**Figure 1:**
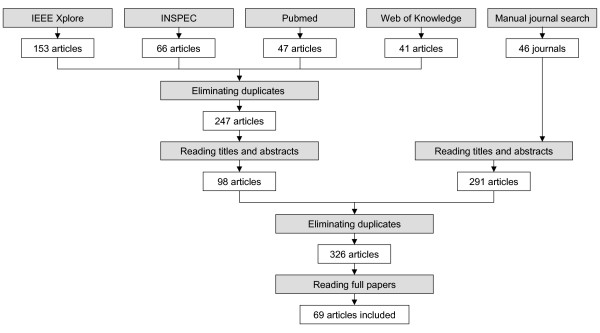
Method used in selecting articles published between 2002 and 2006.

For both the database search and the manual journal search, the titles and abstracts of each article were read by at least two authors, first, to check whether inclusion criteria were met. Dubious articles were not excluded immediately but considered in the second step. Step one resulted in 98 articles from database search and 291 articles from manual journal search. In step two, after duplicates had been eliminated, 326 articles were read in full length, again by at least two authors. In case of any disagreement about inclusion, the respective article was read by a third author who decided about its inclusion or non-inclusion. As illustrated in Figure 1, the final 67 systems described in 69 articles were included in the analysis. In the analysis and discussions below reference is made to the systems, no longer to the articles.

**Figure 2:**
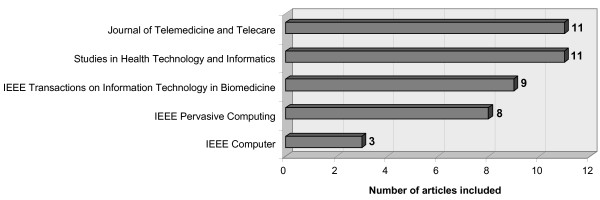
Periodicals including most of the articles selected.

Figure 2 provides an overview of the journals with the largest numbers of selected articles.

Systems and projects were analyzed by the categories of project status, health care setting, users, improvement aims, component types, data gathering, data transmission, systems functions, and deployment issues as well as combinations thereof. For the analyses, the approach of Cruz-Correia et al. [18] was partly adopted, while the definition of categories was partly influenced by other overviews of the topic [7,8,19,20].

## Results

Table 1 displays system and project names, countries of implementation, number of references as well as the actual references. When an article includes two or more systems, the systems are listed separately. When different articles refer to the same system or project, the references are listed together. Finally, 67 distinct systems were identified for the review. The countries with the largest numbers of systems in place are USA (24 systems), UK (8 systems), France (4 systems), Taiwan (4 systems), Australia (3 systems), Denmark (3 systems), Germany (3 systems), Spain (3 systems). A total of 31 systems were implemented in the EU. Two commercial systems are employed internationally.

**Table 1:** Systems included in the review

**System/Project Name**	**Countries**	**Number of Publications**	**Years of Publication**	**References**
ABC (Activity-Based Computing) Framework, Aarhus	Denmark	1	2005	[76]
Activities of Daily Living (ADL) Monitoring System, St. Paul	USA	1	2006	[68]
Activity Tracking and Ambient Displays	USA	1	2003	[21]
Adaptive Coaching through Sequential Routines	USA	1	2003	[21]
Advanced Care and Alert Portable Telemedical Monitor (AMON) Project	Switzerland, Israel, France	2	2004, 2005	[41,42]
Airmed-Cardio System	Spain	1	2005	[50]
Allocation and Group Aware Pervasive Environment (AGAPE) System	Italy	1	2006	[60]
Asset-Tracking System, Durham	USA	1	2003	[56]
Asthma Monitoring System, Oxford	UK	1	2005	[49]
Automated Surveillance System, La Tronche	France	1	2003	[32]
Battlefield Medical Information System-Tactical (BMIST) System	USA	1	2006	[91]
Blood Bag Monitoring System, Shimane	Japan	1	2003	[55]
Care in the Community Project	UK	1	2004	[37]
CareMedia Project	USA	1	2004	[67]
Chronic Care Telemedicine System, Madrid	Spain	1	2006	[88]
Cyber Crumb System	USA	1	2004	[53]
Diabetes Telemedicine System, Oxford	UK	1	2005	[22]
DiaBetNet	USA	1	2004	[57]
Elite Care Business	USA	1	2002	[23]
ENABLE Project, Cooker Monitor	UK, Lithuania, Ireland	1	2004	[72]
ENABLE Project, Night Light	UK, Lithuania, Ireland	1	2004	[72]
Gérontologie Assistée par la Recherche et le Diagnostic des Incidents et des Errances Nocturnes (Gardien) System	France	1	2005	[77]
Hand-Held Decision Support System, Sydney	Australia	1	2005	[78]
Hand-Held Devices in Emergency Department, Western Australia	Australia	1	2004	[83]
Health Feedback Displays	USA	1	2005	[40]
Home Asthma Telemonitoring (HAT) System	USA	1	2004	[48]
Home Automated Telemanagement (HAT) System	USA	1	2006	[26]
Home Monitoring of Implanted Cardioverter Defibrillators, Aachen	Germany	1	2006	[58]
Home-Monitoring System for Cardiac Patients, Graz	Austria	1	2006	[85]
Hospital Ward with Virtual Notes, Trondheim	Norway	1	2006	[74]
Hospital Without Walls Project	Australia	1	2002	[69]
iHospital System, Horsens	Denmark	1	2006	[13]
Implantable Haemodynamic Monitoring System, Minneapolis	USA	1	2005	[59]
Integrated Home Telehealth Care System, Seoul	Korea	1	2005	[45]
Intelligent Emergency Respose (IERS) System	Canada	1	2005	[33]
IST@HOME Project	EU	1	2004	[30]
Karma2 Project	Italy	2	2004, 2005	[51,52]
LifeShirt System	International	1	2004	[46]
MASCAL System	USA	1	2005	[54]
MIThril System	USA	1	2004	[57]
Mobile Emergency Triage (MET) System	Canada	2	2004, 2005	[81,82]
MobileWard System	Denmark	1	2006	[75]
NASA Arrhythmia Monitoring System	USA	1	2004	[24]
Notfall Organisations- und Arbeitshilfe (NOAH) System	Germany	1	2003	[80]
Pervasive Sensor and Activity Tracking (Severe Cognitive Impairment)	USA	1	2003	[21]
Proactive Activity Toolkit (PROACT)	USA	1	2004	[38]
QuietCare System	USA	1	2006	[27]
Real Time Location System, Antwerp	Belgium	1	2006	[39]
Real-Time Wireless Physiological Monitoring System (RTWPMS), Taipei	Taiwan	1	2006	[64]
Remote Monitoring System, Toulouse	France	2	2002	[65,66]
Safety Portal, Taipei	Taiwan	2	2005, 2006	[43,44]
SenseWear System	International	1	2005	[31]
Array-Based Detectors (Simbad) Project	UK	1	2004	[34]
TeleCARE Project	Spain	1	2004	[35]
Triage and Casualty Informatics Technology (TACIT) System	USA	1	2006	[63]
Triage Support System, Taipei	Taiwan	1	2006	[87]
Trinetra System	USA	1	2006	[86]
Tumor Board Project	Germany	1	2006	[79]
Virtual Eye (VI) System	Saudi Arabia, UAE	1	2006	[29]
Wearable Systems in Nursing Home Care, Lulea	Sweden	1	2006	[73]
West Surrey Telemedicine Monitoring Project	UK	1	2003	[62]
Wireless Alerts Pagers, Los Angeles	USA	2	2003, 2005	[70,71]
Wireless Electronic Prescription (EPS) System, London	UK	1	2006	[84]
Wireless Intelligent Sensors (WISE), Huntsville	USA	1	2003	[28]
Wireless Physiological Monitoring, Taipei	Taiwan	1	2004	[61]
Wireless Sensors in Health and Care (WSHC) Project	Norway	1	2006	[47]
Worker Interactive Networking (WIN) Project	USA	1	2006	[36]

### Status of system

Three stages of project status were distinguished: prototype or pilot testing, clinical or medical trials, and regular operation. As shown in Table 2, most systems are presented in their prototype or pilot stages (84%). Authors reported that six systems had passed clinical trials, five systems were found to be in regular operation. This information represents the status as described in the articles, ignoring any subsequent changes.

**Table 2:** Status of systems

**System or project status**	**n (%)**	**References**
Prototype/pilot study	56 (84%)	[13,21,24,26-30,32-45,47-58,60-69,72-77,79,80,83-87]
Clinical trials	6 (9%)	[22,57,59,78,81,82,88]
In regular operation	5 (7%)	[23,31,46,70,71,91]

Note: Systems are assigned to one status category only. The percentages refer to all 67 systems examined.

### Health care settings

The targeted health care settings are differentiated into *ambulatory*, *home and mobile*, *clinical*, *care *and *rehabilitation*. Most systems (57%) are intended for use in home and mobile settings, followed by clinics (36%) (Table 3). Four systems are applied in the ambulatory setting. Seven systems have uses in emergency medical services. Five systems are dedicated to the use in care settings, and no system is explicitly foreseen for rehabilitation.

**Table 3:** Health care settings

**Health care settings**	**n (%)**	**References**
Ambulatory	4 (6%)	[31,46,84,91]
Home/mobile	38 (57%)	[21-24,26-28,30,31,33-38,40-42,45,48-53,57-59,62,65,66,68,69,72,85,86,88]
Emergency	7 (10%)	[24,54,60,63,80,87,91]
Clinical	24 (36%)	[13,29,32,41-44,46,47,53-56,61,64,70,71,74-79,81-83,87,88]
Care	5 (7%)	[39,64,67,69,73]
Rehabilitation	0 (0%)	

Note: Multiple entries in different categories are possible. The percentages refer to all 67 systems reviewed.

### Users

Systems users are divided into *health care professionals*, (i.e. medical personnel, including nurses and professional caregivers, paramedics, physicians) and *lay persons *(i.e. patients and private caregivers, such as family members). Even though, in most cases, several stakeholders profit from an application (e.g., patients benefiting from a better diagnosis by physicians using a system), only the active users or operators were considered as 'users'. As Table 4 shows, nurses and caregivers (51%) and physicians (54%) are nearly equal as designated users. Paramedics are the users of five systems. Not surprisingly, patients are the largest group of users (72%). In many cases, they are supported by lay caregivers or family members involved in nine systems. Four systems involve other user types, i.e. exercise partners in an elderly-care institution [21], pharmacists [22], institution management [23], or a call center [24].

**Table 4:** Users

**Users**	**n (%)**	**References**
Medical professionals		
Nurses/caregivers	34 (51%)	[13,22,23,27,29-31,34,37,39,41-45,47,51,52,54-56,59,62,64-71,73-77,83,87,88]
Paramedics	5 (7%)	[24,54,63,80,91]
Physicians	36 (54%)	[13,22,23,29,31,32,41-52,54,55,58-61,63,64,69-71,74,76,78-85,88,91]
Lay persons		
Patients	48 (72%)	[21-24,26-33,35-54,57-62,64,67-69,72,84-86,88]
Private caregivers/family	9 (13%)	[21,23,30,34-36,40,65,66,72]
Others	4 (6%)	[21,41,68,76]

Note: Multiple entries in different categories are possible. The percentages refer to all 67 systems reviewed.

### Improvement aims

The development and deployment of IT systems in health care is usually driven by intentions to improve medical care or workflow. Therefore, this category is divided into *organizational improvements *(e.g., improved documentation or process automation) and *medical improvements*. Medical improvements are further divided as follows: *Therapy and rehabilitation *deals with situations where the goal is the recovery of the patient, while *prevention and care *encompasses situations where no disease is treated, but a disease or its further progress are to prevented or compensated for. The latter includes care for elderly or support of people with special needs. As Table 5 shows, 39% of all systems seek to improve the organization of health care providers. 12% of all systems were designed to improve therapy and rehabilitation, while 63% seek to enhance prevention and care.

**Table 5:** Improvement aims

**Improvement aims**	**n (%)**	**References**
Organizational improvements	26 (39%)	[13,21,23,29,39,43,44,47,54-56,63,70,71,73-76,78-84,87,91]
Medical improvements		
Therapy and rehabilitation	8 (12%)	[22,31,46,50-52,57,85]
Prevention and care	42 (63%)	[21,23,24,26-42,45,46,48,49,53,58-62,64-69,72,77,86-88]

Note: Multiple entries in different categories are possible. The percentages refer to all 67 systems reviewed.

In addition, medical improvements are categorized according to the *body subsystem and disease *categorization of the Medical Subject Headings of the U.S. National Library of Medicine [25]. Categories encompass the cardiovascular system, respiratory tract, endocrine system, sensory organs, nervous system, and others. These categories were selected according to the diseases mentioned in the studies included. A large part of the systems refer to the nervous system (21%), dementia being mentioned most often (Table 6). In addition, 18% of the systems refer to the cardiovascular system, in particular to heart arrhythmia or chronic heart diseases. Eight systems target the respiratory tract, with chronic obstructive pulmonary disease (COPD) as the most important case. Two systems are dedicated to diabetes treatment (endocrine system). The seven systems in the 'Others' category cover, for instance, inflammatory bowel disease [26], cancer [27], or stress [28]. Also, 24% of systems are found to have no specific targeted disease or part of the body subsystem. In many cases, systems monitor multiple physiological parameters for diverse health care applications. Other systems provide general information about the status of patients or inhabitants [23,29-31] or monitor presence, movements or behavioral patterns of residents of care institutions [23,32-40]. The 'Data gathering' Section below provides more details on the different types.

**Table 6:** Medical improvements by body subsystems

**Body Subsystems**	**n (%)**	**References**
Cardiovascular System	12 (18%)	[24,28,41,42,45,46,50,58-61,64,85]
Respiratory Tract	8 (12%)	[41,42,45,46,48,49,62,88,91]
Endocrine System	2 (3%)	[22,57]
Sensory Organs	1 (1%)	[86]
Nervous system	14 (21%)	[21,46,51-53,57,65-67,72,77,86,91]
Others	7 (10%)	[26-28,46,57,68,91]
No specific disease	16 (24%)	[23,29-40,43,44,69,87]

Note: Multiple entries in different categories are possible. The percentages refer to all 67 systems reviewed.

### Systems features

Four variables which characterize specific systems features, i.e. component types, types of data gathering, data transmission, and systems functions, were selected.

#### Component types

Systems are classified into those with mobile and stationary components. Systems with mobile components were differentiated as *conventional mobile devices*, *wearables*, and *implanted devices*. *Stationary devices *are computer-enhanced physical environments, such as buildings or furniture. As depicted in Table 7, 51% of systems are found to utilize conventional mobile devices. Stationary devices are used equally often (51%), in many cases in a comprehensive, integrated application of systems, such as information exchange systems in hospitals or for monitoring in care facilities. A considerable fraction use wearables (42%) including wrist-worn units [31,41-45], an electronic vest [46], an electronic glove [38] as well as mobile medical devices, such as a blood glucose meter [22], blood pressure meter [47], spirometer [48], asthma peak flow meter [49], electrocardiogram (ECG) or heart rate monitors [24,28,50], or multi-purpose meters [30,51,52]. Also electronic person tags or badges [21,23,39,53,54], electronic object tags [21,38,55,56] or a customizable modular system [57] belong to this component type. Only two systems have implanted devices, i.e. a cardiac pacemaker with a monitoring function [58] and an implantable haemodynamic monitoring system [59].

**Table 7:** Component types

**Component types**	**n (%)**	**References**
Conventional mobile device	34 (51%)	[13,22,26,28-30,36,40,43,44,48-50,54,57,60,61,63,64,69-71,73-76,78,80-88,91]
Wearables	28 (42%)	[21-24,28,30,31,38,39,41-57,60,62,73]
Implanted devices	2 (3%)	[58,59]
Stationary devices	34 (51%)	[13,21,23,27,28,30,32-40,45,53,59,61,64-69,72,74-77,79,84]

Note: Multiple entries in different categories are possible. The percentages refer to all 67 systems reviewed.

#### Data gathering

The systems are classified by five types of data gathering or data input: *monitoring of persons or objects; localization of persons or objects *as well as *manual input or request *by the user. As presented in Table 8, most systems monitor persons (63%), typically by gathering physiological or behavioral data. For physiological data or vital signs, respectively, systems range from measuring a single physiological parameter, i.e. ECG [24,50,60], lung function (asthma) [48,49], haemodynamic trends [59], blood glucose (diabetes) [22,57], heart rhythm [58], blood pressure [47], or weight [26], to simultaneously gathering multiple physiological data [28-31,42,45,46,51,52,61-64].

**Table 8:** Types of data gathering

**Type of data gathering**	**n (%)**	**References**
Monitoring of persons	42 (63%)	[21-24,26-31,33-36,39-42,45-53,57-69,88,91]
Monitoring of objects	11 (16%)	[21,27,40,43,44,55,68,70-72,79,86]
Localization of persons	21 (31%)	[13,23,24,27,32,35,37,39,40,43,44,53,54,60,63,67,68,73-77]
Localization of objects	3 (4%)	[38,54,56]
Manual input or request	19 (28%)	[40,48,50,59,62,63,73-76,78-87]

Note: Multiple entries in different categories are possible. The percentages refer to all 67 systems reviewed.

Behavioral data gathering includes monitoring of presence, movements or activities [21,35,37,65,66], such as monitoring of 'Activities of Daily Living' (ADL) [38,67,68], sleeping or overnight activities [27,41], medication adherence [27], presence or movements in rooms or facilities [21,36,39,53], social or communicative behavior [40], or detection of falls [33,34]. Also combinations of monitoring multiple physiological parameters and movement data can be found [23,69]. In one case, the purpose of person identification is mentioned explicitly [67]. On the other hand, monitoring of objects is less frequent (16%). It includes, for instance, RFID-based inventory control [43], monitoring of blood bag temperature [55], checking for lab results [70,71] or monitoring conditions or activities of persons, such as indicating sleeping conditions by bed sensors [68,72].

The second most frequent type of data input is localization of persons (31%). This encompasses the localization of medical personnel within hospitals or care facilities, in most cases, for context-aware or location-dependent information [13,73-76]. Most systems focus on the localization of patients or residents within facilities [23,27,32,35,37,39,40,43,44,63,67,68,77], or in larger geographical areas by GPS [24]. Localization is also used to support persons with special needs, for instance, for directing blind persons [53], or to assist ad hoc groups of helpers in emergency situations [60]. Only one system furnishes multiple localizations of personnel, patients, and equipment [54] while three systems localize objects, i.e. medical equipment [54,56] or RFID-tagged objects of daily life allowing conclusions to be drawn about activities of the persons monitored [38].

A large percentage of systems require manual input or request of data (28%), mainly in mobile devices, such as PDAs or tablet PCs. The data is entered by health care or care personnel [63,74,75,78-84] or patients [48,50,85], residents of care facilities [40] or people with special needs [86]. In many cases, manual input or request is an additional channel besides automatic monitoring or localization. For instance, manual involvement consists of transmitting parts of the physiological parameters or supportive results of questionnaires [50], supportive telephone calls by a nurse parallel to automatic transmission of monitoring data [62], or data access through a web interface [59]. In other cases, speech recognition is supported by manual data input [73,87].

#### Data transmission

There are systems *transmitting data *to other systems or players and those which do not. With data transmission, the data leave the area of control by a specific user, which may have implications on privacy and security (see below). As is shown in Table 9, most systems transmit data (88%), for example, for purposes of data analysis, forwarding, or storage. In many cases, data are transmitted to a central server. All systems developed for data exchange among multiple users depend – by nature – on data transmission. About 19% of all systems do not rely completely on data transmission and are able to perform functions independently and in a decentralized fashion. About half of those systems consist of wearables monitoring patient health or activity [31,38,42,45,57], assisting the user by providing supportive information about the health status or by suggesting certain activities. Some systems can perform parts of their functions both with and without data transmission [31,40,41,45,48,57,72].

**Table 9:** Data transmission

**Data transmission**	**n (%)**	**References**
With data transmission	59 (88%)	[13,21-24,26-31,34-36,39-77,79-86,88,91]
Without data transmission	13 (19%)	[21,31-33,38,40-42,45,48,57,72,87]
Not described well enough	2 (3%)	[37,78]

Note: Multiple entries in different categories are possible. The percentages refer to all 67 systems reviewed.

#### Systems functions

The functions provided by the systems are subdivided into six categories: *analytical and diagnostic support*; *alerting*; *medical treatment*; *support activities *(e.g., reminding or guidance); *process automation*; and *documentation and information*. As is evident from Table 10, about 60% of all systems provide analytical and diagnostic functions, often in combination with automatic alerting, which is performed by 46% of all systems. Most of these systems perform physiological monitoring, in particular of the cardiovascular system [28,29,41,50,61]. There are other disease-specific systems, for example, for diabetes [22,48] or asthma [49,57]. A large percentage of systems do not perform physiological monitoring, but obtain analytical and diagnostic support from tracking the activities or behavior of patients [27,65,67-69,77]. The 'support activities' category (34%) includes heterogeneous functions, such as providing reminders for medication [85], scheduling for social contacts [21], orientation in buildings [53], or a product bar code translation for blind people [86].

**Table 10:** Systems functions

**Functions**	**n (%)**	**References**
Analytical and diagnostic support	40 (60%)	[22-24,26-32,35,37,41,42,45-52,57-62,64-69,77,79-82,85,87,88,91]
Alerting	31 (46%)	[13,21-24,27,29,30,33-36,39,41-45,47,48,58,60-62,64,68-72,77,83,85,88]
Support activities	23 (34%)	[13,21-23,26,30,35,38,40,45,48,53,57,60,70-74,76,81,82,85,86]
Information and documentation	21 (31%)	[13,23,27,43,44,46,48,54,55,61,63,64,74-76,78-83,88,91]
Process automation	11 (16%)	[13,39,43,44,55,56,73,76,79,81,82,84,91]
Medical treatment	0	

Note: Multiple entries in different categories are possible. The percentages refer to all 67 systems reviewed.

Documentation and information is a function in 31% of all systems. It includes systems providing context-aware information about patient data and laboratory reports during surgical interventions [13,79] or in morning rounds [76]. Many systems supporting the emergency triage process store data for documentation purposes [63,80-82]. Several systems measuring physiological parameters also store data for documentation [46,48,61,88]. One system provides trend information about the behavior of elderly people receiving care [27].

About 16% of all systems target process automation, the most important task of which is to tracking persons [13,39,73,76], or inventories [56]. Identification of persons via RFID [43,44], phone call interception [73] or electronic prescription transmission [84] are other examples in this category. No system is dedicated to medical treatment, which could be conceivable as computer-supported and remote medication.

### Analysis of health care settings

Combinations of the categories health care settings, improvement aims, functions, and component types also provide *cross-category analyses *in order to gain some insight into the use of systems in different health care settings. For these cross-category analyses, it must be pointed out that the allocations of systems refer to their full functionalities. Thus, multiple entries under different combinations are possible.

#### Health care settings and improvement aims

Not surprisingly, organizational improvements, achieved mostly by automation of manual activities, are the primary goal in the clinical setting (24%), followed by improved prevention and care (15%) (Table 11). In the home and mobile settings, however, the systems primarily seek to provide medical improvements mainly in prevention and care (48%) followed by therapy and rehabilitation (10%). Here, only four systems are to achieve organizational improvements.

**Table 11:** Health care settings and improvement aims

		**Health care settings**				
		
		**Ambulatory**	**Home/mobile**	**Clinical**	**Care**	**Emergency**
**Organizational improvements**	**n (%)**	2 (3%)	4 (6%)	16 (24%)	2 (3%)	5 (7%)
	**References**	[84,91]	[21,23]	[13,29,43,44,47,54-56,70,71,74-76,78,79,81-83,87]	[39,73]	[54,63,80,87,91]

**Medical improvements**						

**Therapy and rehabilitation**	**n (%)**	2 (3%)	7 (10%)	1 (1%)	0	0
	**References**	[31,46]	[22,31,50-52,57,85]	[46]		
**Prevention and care**	**n (%)**	2 (3%)	32 (48%)	10 (15%)	4(6%)	3 (4%)
	**References**	[31,46]	[21,23,24,26-28,30,31,33-38,40-42,45,48,49,53,58,59,62,65,66,68,69,72,86,88]	[29,32,41,42,46,53,61,64,77,87,88]	[39,64,67,69]	[24,60,87]

Note: Multiple entries in different categories are possible. The percentages refer to all 67 systems reviewed.

#### Health care settings and systems functions

Table 12 sheds some light on the ways systems functions are used in the respective health care settings. As expected, analytical and diagnostic support (18%), alerting (14%), and support activities (11%) are most popular in home or mobile settings whereas information and documentation (four systems) or process automation and control (no system) play only a negligible role. For clinical applications, the systems functions are distributed more evenly, information and documentation (10%) being most popular.

**Table 12:** Health care settings and functions

**Functions**		**Health care settings**				
		
		**Ambulatory**	**Home/mobile**	**Clinical**	**Care**	**Emergency**
**Analytical and diagnostic support**	**n (%)**	3 (2%)	26 (18%)	12 (8%)	3 (2%)	5 (3%)
	**References**	[31,46,91]	[22-24,26-28,30,31,35,37,41,42,45,48-52,57-59,62,65,66,68,69,85,88]	[29,32,41,42,46,47,61,64,77,79,81,82,87,88]	[64,67,69]	[24,60,80,87,91]
**Alerting**	**n (%)**	0	20 (14%)	11 (7%)	3 (2%)	2 (1%)
	**References**		[21-24,27,30,33-36,41,42,45,48,58,62,68,69,72,85,88]	[13,29,41-44,47,61,64,70,71,77,83,88]	[39,64,69]	[24,60]
**Support activities**	**n (%)**	0	16 (11%)	6 (4%)	1 (1%)	1 (1%)
	**References**		[21-23,26,30,35,38,40,45,48,53,57,72,85,86]	[13,53,70,71,74,76,81,82]	[73]	[60]
**Information and documentation**	**n (%)**	2 (1%)	4 (3%)	15 (10%)	1 (1%)	4 (3%)
	**References**	[46,91]	[23,27,48,88]	[13,43,44,46,54,55,61,64,74-76,78,79,81-83,88]	[64]	[54,63,80,91]
**Process automation and control**	**n (%)**	2 (1%)	0	7 (5%)	2 (1%)	1 (1%)
	**References**	[84,91]		[13,43,44,55,56,76,79,81,82]	[39,73]	[91]

Note: Multiple entries in different categories are possible. The percentages refer to all 67 systems reviewed.

#### Health care settings and component types

In Table 13, it can be seen that components implemented most frequently in home or mobile setting are stationary devices (33%) and wearables (28%), followed by conventional mobile devices (21%). In the clinical setting, conventional mobile devices are most prevalent (22%), followed by stationary devices (15%), while eight systems make use of wearables. There is no system implementing stationary devices in emergency settings, as most of the systems take advantage of conventional mobile devices.

**Table 13:** Health care settings and component types

		**Health care settings**				
**Component types**		**Ambulatory**	**Home/mobile**	**Clinical**	**Care**	**Emergency**

**Conventional mobile devices**	**n (%)**	2 (3%)	14 (21%)	15 (22%)	3 (4%)	6 (9%)
	**References**	[84,91]	[22,26,28,30,36,40,48-50,57,69,85,86,88]	[13,29,43,44,54,61,64,70,71,74-76,78,81-83,87,88]	[64,69,73]	[54,60,63,80,87,91]
**Wearables**	**n (%)**	2 (3%)	19 (28%)	8 (12%)	2 (3%)	3 (4%)
	**References**	[31,46]	[21-24,28,30,31,38,41,42,45,48-53,57,62]	[41-44,46,47,53-56]	[39,73]	[24,54,60]
**Implanted devices**	**n (%)**	0	2 (3%)	0	0	0
	**References**		[58,59]			
**Stationary devices**	**n (%)**	1 (1%)	22 (33%)	10 (15%)	4 (6%)	0
	**References**	[84]	[21,23,27,28,30,33-38,40,45,53,59,65,66,68,69,72]	[13,32,53,61,64,74-77,79]	[39,64,67,69]	

Note: Multiple entries in different categories are possible. The percentages refer to all 67 systems reviewed.

### Analysis of systems features

#### Systems functions and types of data gathering

Table 14 provides information about the types of data gathering approaches enabling the respective functions of the systems. Monitoring of persons is by far the most important type of data gathering for analytical and diagnostic support (48%), alerting (36%) as well as support activities (19%), followed by localization of persons. Monitoring and localization of objects generally occur less frequently and are applied in typical activities for organizational improvements, such as information and documentation as well as process automation and control. For all functions, very many systems require manual data input, which could indicate that complete automation may not yet be fully possible or desirable.

**Table 14:** Systems functions and data gathering

		**Systems functions**				
**Data gathering**		**Analytical and diagnostic support**	**Alerting**	**Support activities**	**Information and documentation**	**Process automation and control**

**Monitoring of persons**	**n (%)**	32 (48%)	24 (36%)	13 (19%)	9 (13%)	2 (3%)
	**References**	[22-24,26-31,35,41,42,45-52,57-62,64-69,88,91]	[21-24,27,29,30,33-36,39,41,42,45,47,48,58,60-62,64,68,69,88]	[21-23,26,30,35,40,45,48,53,57,60]	[23,27,46,48,61,63,64,88,91]	[39,91]
**Monitoring of objects**	**n (%)**	3 (4%)	5 (7%)	5 (7%)	4 (6%)	3 (4%)
	**References**	[27,68,79]	[27,43,44,68,70-72]	[21,40,70-72,86]	[27,43,44,55,79]	[43,44,55,79]
**Localization of persons**	**n (%)**	10 (15%)	10 (15%)	9 (13%)	9 (13%)	5 (7%)
	**References**	[23,24,27,32,35,37,60,67,68,77]	[13,23,24,27,35,39,43,44,60,68,77]	[13,23,35,40,53,60,73,74,76]	[13,23,27,43,44,54,63,74-76]	[13,39,43,76,44,73]
**Localization of objects**	**n (%)**	0	0	1 (1%)	1 (1%)	1 (1%)
	**References**			[38]	[54]	[56]
**Manual input or request**	**n (%)**	9 (13%)	4 (6%)	8 (12%)	10 (15%)	5 (7%)
	**References**	[48,50,59,62,79-82,85,87]	[48,62,83,85]	[40,48,73,74,76,81,82,85,86]	[48,63,74-76,78-83]	[73,76,79,81,82,84]

Note: Multiple entries in different categories are possible. The percentages refer to all 67 systems reviewed.

#### Systems functions and component types

Table 15 illustrates that there is no one particular type of component dominating implementation of analytical and diagnostic support as well as alerting and support activities, although there is a slight prevalence of conventional mobile devices for analytical and diagnostic support as well as of stationary devices for alerting. Differences are found for functions aimed at achieving organizational improvements. In this case, conventional mobile devices are most popular for information and documentation as well as for process automation and control.

**Table 15:** Systems functions and component types

		**Systems functions**				
**Component types**		**Analytical and diagnostic support**	**Alerting**	**Support activities**	**Information and documentation**	**Process automation and control**

**Conventional mobile devices**	**n (%)**	19 (28%)	15 (22%)	15 (22%)	16 (24%)	7 (10%)
	**References**	[22,26,28-30,48-50,57,60,61,64,69,80-82,85,87,88,91]	[13,22,29,30,36,43,44,48,60,61,64,69-71,83,85,88]	[13,22,26,30,40,48,57,60,70,71,73,74,76,81,82,85,86]	[13,43,44,48,54,61,63,64,74-76,78,80-83,88,91]	[13,43,44,73,76,81,82,84,91]
**Wearables**	**n (%)**	18 (27%)	13 (19%)	11 (16%)	6 (9%)	5 (7%)
	**References**	[22-24,28,30,31,41,42,45-52,57,60,62]	[21-24,30,39,41-45,47,48,60,62]	[21-23,30,38,45,48,53,57,60,73]	[23,43,44,46,48,54,55]	[39,43,44,55,56,73]
**Stationary systems**	**n (%)**	17 (25%)	17 (25%)	13 (19%)	9 (13%)	5 (7%)
	**References**	[23,27,28,30,32,35,37,45,59,61,64-69,77,79]	[13,21,23,27,30,33-36,39,45,61,64,68,69,72,77]	[13,21,23,30,35,38,40,45,53,72,74,76]	[13,23,27,61,64,74-76,79]	[13,39,76,79,84]

Note: Implanted devices are not included in the table. Multiple entries in different categories are possible. The percentages refer to all 67 systems reviewed.

#### Systems flexibility and complexity

Next, the *flexibility of the systems *will be analyzed in the light of whether a particular system performs more than one system function. As is shown in Table 16, 30 systems (45%) are identified which perform one function only: analytical and diagnostic support (12 systems), support activities (7 systems), alerting (5 systems), information and documentation (4 systems), and process automation and control (2 systems). As functions increase, systems naturally become more complex while the number of systems actually capable of performing multiple functions decreases. This analysis revealed 19 systems performing two functions, 14 systems with three functions, and four systems able to perform four functions.

**Table 16:** Systems flexibility

	**n (%)**	**References**
**One-function systems**	30 (45%)	
Analytical and diagnostic support	12 (18%)	[28,31,32,37,49-52,57,59,65-67,87]
Alerting	5 (7%)	[21,33,34,36,72]
Support activities	7 (10%)	[21,38,40,53,72,86]
Information and documentation	4 (6%)	[54,63,75,78]
Process automation and control	2 (3%)	[56,84]
**Two-function systems**	19 (28%)	[24,26,29,39,41,42,46,47,55,57,58,62,68-71,73,74,77,80,83]
**Three-function systems**	14 (21%)	[22,27,30,35,43-45,60,61,64,76,79,85,88,91]
**Four-function systems**	4 (6%)	[13,23,48,81,82]

Note: No multiple entries. The allocation of systems is unique.

In the context of this study, *systems complexity *is expressed by the number of different types of components in a system. A distinction is made between systems using a single device or multiple devices made up of one (57%) or more than one component type (Table 17). Among these systems using only one type of component, stationary devices are most frequent (21%), mainly as comprehensive ICT infrastructure embedded in facilities. They are closely followed by conventional mobile devices (19%) and wearables (15%). In the category of systems combining two component types (40%), the combination of conventional mobile devices with stationary devices and wearables was found most frequently (10 and 9 systems, respectively), followed by the combination of wearables and stationary devices (7 systems). Only two systems were found to implement as many as three different types of components.

**Table 17:** Systems complexity

	**n (%)**	**References**
**Single-component system**	38 (57%)	
Conventional mobile devices	13 (19%)	[26,29,63,70,71,78,80-83,85-88,91]
Wearables	10 (15%)	[24,31,41,42,46,47,51,52,55-57,62]
Implanted devices	1 (1%)	[58]
Stationary devices	14 (21%)	[21,27,32-35,37,65-68,72,77,79]
**Two-component system**	27 (40%)	
Conventional mobile devices and wearables	9 (13%)	[22,43,44,48-50,54,57,60,73]
Conventional mobile and stationary devices	10 (15%)	[13,36,40,61,64,69,74-76,84]
Wearables and stationary devices	7 (10%)	[21,23,38,39,45,53]
Implanted and stationary devices	1 (1%)	[59]
**Three-component system**		
Conventional mobile and stationary devices and wearables	2 (3%)	[28,30]

Note: No multiple entries. The allocation of systems is unique.

### Deployment issues

Under this heading, the focus is on *organizational or personnel issues*, *privacy and security issues*, and *financial issues*. Although these issues are crucial for the success of pervasive computing in health care, they are rarely addressed in the literature (Table 18). Hence, no quantitative analysis of these topics was possible. The qualitative analysis shown below revealed some interesting findings in articles about issues of deployment.

**Table 18:** Deployment issues

**Deployment issues**	**References**
Organizational or personnel issues	[13,47,56,69-71,74]
Financial issues	[13,30,34,36,39,53,55,56,86,88]
Privacy, security, and control issues	[13,24,34,41,42,50,54,56,67,83,84,88]
No deployment issues	[23,26,28,29,32,33,35,37,38,41-44,57,61,64-66,68,80,87]

Note: Multiple entries in different categories are possible.

#### Issues of organization and personnel

Issues of organization or personnel are described for only six systems (Table 18). Positive experiences are reported from an interpersonal communication service utilizing digital notes within hospital wards [74]. The author investigates the integration of the system into regular clinical activities. One of the findings is that the system is better adapted to a high degree of mobility and the highly event-driven working patterns of clinicians than conventional communication technology, such as telephone or fax. Among other things, digital notes have the advantage of not interrupting work routines and providing information in a use context.

The need for organizational issues to be separated into home applications and applications in clinics is stressed by Dadd et al. in a study of a monitoring system [69]. While, in a home setting, monitoring is a long-term procedure relatively unattended, monitoring in clinics takes place in an environment where many health professionals, technical assistance and substitute equipment are available. The organizational difference leads to varying technical design requirements.

Other authors write about organizational problems to be solved for successful deployment and regular operation. For the implementation of a wireless biomedical sensor for blood pressure measurement during surgery, Øyri et al. [47] concluded that nursing education should include a stronger focus on nursing informatics. Since nurses played a role in protecting patients, better education could help them overcome potentially conservative attitudes toward change.

Organizational problems with alert pagers in a surgical intensive care unit are examined by Reddy et al. [70]. The pager automatically alerts about critical lab results, potential medication problems, and critical patient trend information. One problem is that every message is sent not only to residents and fellows, but simultaneously to physicians who normally would be informed only about important clinical events. The removal of hierarchical boundaries by providing information to everyone, thus, also has unintended negative consequences. Moreover, the unidirectional nature of pagers prevents physicians from responding to a problem in the same way. Physicians also complain of information overload, as every message looks equally important. Another organizational problem is that nurses, who are responsible for supplying physicians with adequate information, do not know whether physicians are already aware of information sent automatically. Thus, nurses might additionally inform physicians about events. Several technical measures are proposed by the authors to better match organizational needs.

Hansen et al. [13] describe organizational issues which emerged in the deployment of the iHospital system, i.e. a hospital scheduling and awareness system. The system utilizes location tracking, video streaming for context information, large interactive displays, and mobile phones. In order to teach users, some of them are familiarized with the system, encouraged in using it, and asked to pass on information and experience to others. The project team also reported that systems causing extra workload and mainly benefiting others are likely to be not used. One example is a tracking chip requiring daily pickup and registration. They also point to the problem of the missing definition of who is responsible for occasionally time consuming and complex systems support after deployment. A similar case is presented by Østbye et al. [56] as result of focus groups involving nursing staff. Nurses raised concerns about the additional workload due to the implementation of new systems, especially when a system is not easy to use or no additional staff is hired for systems maintenance.

#### Privacy and security issues

Authors report about privacy, security and control issues for 11 systems (Table 18), in some cases only by mentioning that appropriate technical measures ensure systems compliance with data protection laws. Such technical measures include the removal of user identity from data [41] and encryption and authentication steps prior to data transmission as part of the GSM/GPRS protocol [41,42], with a secure WAP session [50], or with session key encryption and digital signing using a public key certificate [84]. Another study of a telemedicine system for COPD implements a broad set of security measures ranging from password log-ins, PKI certificates, tokens, SSL encryptions, VPN to restricting use only to the intranet [88]. In another case, it is noted that the encryption level of the tags used to track patients, equipment and staff is too weak for regular operation [54]. To control access to stored information, one system includes a set of layers with different access privileges for different user groups [50]. In a trial of a hand-held computing device in an emergency department, data security was to be achieved by a policy requiring that no patient data be stored on the hand-held device and be deleted immediately after transmission to a server. In addition, the system sends an alert to security personnel when the hand-held device crosses certain facility boundaries [83].

As reported by Hansen et al. [13] for the implementation of the iHospital system, the project team found less privacy concerns among participants than expected, although privacy-sensitive data from video multicast and location tracking are processed. The authors had concluded from interviews and observation that the users would trust the system because of the chosen design, in particular, the low-resolution video streams and only partial location tracking, which left 'tracking free' areas, such as the coffee room, cafeteria, and bathrooms. The issue of surveillance of medical staff is also mentioned by Østbye et al. [56] in a case study of an equipment tracking system. In that case, nursing staff voiced concerns about surveillance of their work patterns once the system would be used more widely.

Hauptmann et al. [67] present a pervasive computing system for elderly care which is able to track people over long periods of time, identify individuals, and characterize human activities, such as eating or personal hygiene. In the opinion of Hauptman et al., activity observation and detection is feasible as long as the benefits of monitoring for care purposes are not outweighed by privacy concerns.

Sixsmith et al. [34] provide some findings by focus groups involving users of infrared monitoring systems for detecting falls in an elderly-care setting, in which concerns about intrusiveness are raised. The authors report that lack of understanding the technology is partly responsible for privacy concerns, as the system would not be able to reconstruct an image for viewing. They conclude that adequate information about technology is important during deployment. "Whatever the practical benefits might be, users might not accept the technology if they believe it impinges on their privacy and lifestyle" [34].

In one case study [24], the authors clearly state that the treatment of location data – in this case, a real-time remote heart arrhythmia monitoring system – is an unsolved privacy issue. Recommendations for the appropriate collection, use, and retention of these data are still missing (e.g., the frequency of location data acquisition and transmission, or coordinate accuracy). The authors describe the potential of misuse stemming, in particular, from combinations of databases containing heart health indicators with continuous, time-stamped location data. The authors conclude that telemedicine community and medical community should participate in defining privacy-related rules and guidelines. They also point to another unsolved dilemma: encryption of data for transmission could "... sacrifice precious minutes during heart attack" [24].

#### Financial issues

Authors mention financial issues for only ten systems (Table 18). No author provides a profound analysis of costs and benefits in economic terms. The most detailed analysis is by Østbye et al. [56] for an equipment tracking system for beds, sequential compression devices, and infusion pumps. They report about the impact on equipment use and, thus, equipment charge capture, but show no positive results for all objects tracked.

Some case studies only provide rough estimations or brief notes, such as reference to the participation of business [36] or to an expected large market for the system [34]. For a telemedicine system for COPD, de Toledo et al. [88] present a rough estimate to the effect that significantly shorter hospitalization would lead to fast amortization of the system. Some case studies only mention the costs of system components [39,53] indicating that scaling up to regular widespread operation would change the cost structure (e.g., due to other license fees with more users) [39]. Authors also assume that changes in reimbursement systems, such as the introduction of the Diagnosis Related Groups system, would have a considerable impact on finance [30].

The case studies also contain information about possible cost reductions. Hanada et al. [55] estimate that only substantial price reductions of blood bag tags would make the system profitable. The potential saving of financial losses in a year due to inappropriate temperature management then would offset the cost of the entire blood bag monitoring system. Cost reductions are expected to arise in particular from the use of commercial off-the-shelf technologies. As described by Narasimhan [86], the Trinetra system, which supports blind shoppers in reading product bar codes in stores, is a result of costs considerations. It utilizes off-the-shelf technology, i.e. a mobile phone, a bar code scanning pen, and a Bluetooth headset. As no investment is required from store owners, the chance that the system will be used regularly is considerably better. Hansen et al. [13] report about the high costs of a commercial location tracking system, which would make the solution financially unattractive for the entire hospital. Therefore, they establish their location tracking technology on the basis of mobile phones owned by physicians or patients.

## Discussion

Although forgoing mere descriptions of systems architectures or concepts and focusing instead on prototypes, experiments, pilot studies, clinical trials – involving intended end users – as well as systems already in regular operation, the articles cover less deployment issues than expected. The partly qualitative analysis thus can only indicate potential deployment problems requiring further consideration. We assume that many authors do not wish to report on critical or negative issues. However, as most systems identified were in the prototype stage, such experiences would be particularly valuable in order to leverage pervasive computing and transfer more systems into routine operation.

Findings in the case studies revealed, for instance, that privacy protection is not only an issue in the relation between health care provider and patient – which currently is the main focus of academic and public debates -, but is also an internal concern of health care staff. When coupled with organizational and personnel concerns, pervasive computing innovations could well be stifled by staff worries about surveillance. Also, the regulatory framework for reimbursement and financing can be decisive for pervasive computing systems to be accepted in routine use. There are also indications that the use of off-the-shelf technologies is promising because of possible costs reductions. Although 34% of the systems reviewed already use conventional mobile devices, we expect their number to increase further, as these devices are becoming more and more powerful.

The case studies also reveal that developers often think about technical measures to protect privacy. We consider institutional measures and policies as equally crucial not only for individual acceptance but for societal acceptability [89]. This latter distinction is crucial, since the individual acceptance of privacy-related applications, for example, by a suffering patient or a dependent health care employee is expected to be much greater than the level of acceptability demanded by society and its representatives in balanced decisions. This is relevant, for instance, to determine the point where the benefits of monitoring are outweighed by threats to privacy protection. This point should be defined in a general manner by guidelines and policies and cannot be determined in case studies involving patients or employees. General binding decisions about the types of data gathered by pervasive computing applications as well as rules about where the data are used, by whom and in what form, should be developed, communicated, adopted, and enforced. Pervasive computing may imply that more players are involved in care relations and the management of personal data, such as systems or service providers, relatives, or multiple medical or care providers in complex health care situations, such as integrated health care.

In the case studies examined, privacy-enhancing technical measures mostly have an 'add-on' character, such as encryption added to data transmission. Instead, a number of systems do not require data transmission to other systems or players. Data transmission involves other parties, thus inevitably raising privacy concerns. Approaches to 'self-supported' [17] pervasive computing requiring neither data transmission nor the use of central and external hardware and software infrastructures are an interesting option in personal health care and personal support of health care staff. For instance, some of the systems mentioned above provide supportive information about health status or make certain suggestions without including data transmission [31,38,42,45,57]. This can contribute to the development of 'persuasive computing' in health care [90]. Further technological developments, particularly improvements in data storage and processing capabilities of mobile devices and wearables, can accomplish pervasive computing without data transmission. These will enable a shift of analytical, alerting, guiding and other functions from central servers to mobile and wearable devices. Less user worry about surveillance could be a result.

### Limitations

There is a gap between the systems we analyzed in our literature review and the full field of pervasive computing in health care. First, many systems developments and implementations might never have undergone scientific peer-review and, therefore, can not be covered by our review of the literature. Second, being limited to the period of 2002 to 2006, the literature studied does not incorporate the most recent developments in computer science, medical informatics, and engineering. In particular, progress in mobile computing technology is substantial, for instance in location-based services. We are aware also of the prevalent time lags between system development, system description, submission and publication of articles.

Our approach does not allow drawing conclusions on whether the results presented in this review are altered by technological progress. In addition, we can not conclude whether the results would be similar for systems not covered by the literature. Therefore, a generalization of conclusions drawn from this literature review is not possible; all conclusions discussed are drawn from the case studies reviewed.

Furthermore, although the inclusion criteria for systems mentioned above are broadly defined and based on previous research, other pervasive computing experts, authors, users etc. may regard other features as decisive.

## Conclusion

This review provides an overview of the literature on a broad and heterogeneous range of pervasive computing systems related to health care. Most systems are described in their prototype stages in which developers only rarely report about deployment issues. Since the identifying and solving such issues is decisive for the diffusion of promising systems, a need for further focused research into the deployment of pervasive computing systems in health care is identified. Future research should focus on organizational as well as personnel implications, privacy concerns or financial issues. Systematic evaluations of the effectiveness and efficiency of pervasive computing systems are regarded as inevitable to ensure user acceptance, societal acceptability and financing.

## Competing interests

The authors declare that they have no competing interests.

## Authors' contributions

CO, AG, and TF contributed equally to the study design, literature search, reading, categorizing and analyzing. CO and AG prepared the manuscript, while TF revised it critically. CO was the initiator of the literature review and additionally performed supervisory tasks. All authors read and approved the final manuscript.

## Pre-publication history

The pre-publication history for this paper can be accessed here:



## Supplementary Material

Additional file 1 **List of journals included in the manual search**. The additional "List of journals included in manual search.pdf" PDF file contains journals searched manually in the literature review. The file lists journal names and links to their websites. Where available, further links to PubMed references as well as Open Access versions of journals at PubMed Central (PMC) are provided. Click here for file
