# Supplementary material for: Towards pervasive computing in health care – A literature review
Source: BMC Med Inform Decis Mak. 2008 Jun 19;8:26. doi: 10.1186/1472-6947-8-26 (PMC2467411; doi:10.1186/1472-6947-8-26)
Supplement: Additional file 1 — List of journals included in the manual search. The additional "List of journals included in manual search.pdf" PDF file contains journals searched manually in the literature review. The file lists journal names and links to their websites. Where available, further links to PubMed references as well as Open Access versions of journals at PubMed Central (PMC) are provided. [file 1472-6947-8-26-S1.pdf]

List of journals included in the manual search

Journal name	Website	PubMed	PMC
AMIA Annual Symposium Proceedings	Website	References	Open Access
Annual Review of Information Science and Technology	Website	no	no
Artificial Intelligence in Medicine	Website	References	no
BMC Medical Informatics and Decision Making	Website Open Access	References	Open Access
CIN: Computers, Informatics, Nursing	Website	References	no
Computer Methods and Programs in Biomedicine	Website	References	no
Computers in Biology and Medicine	Website	References	no
Disease Management and Health Outcomes	Website	no	no
Electronic Journal of Health Informatics	Website Open Access	no	no
Health Informatics Journal	Website	References	no
Health Information and Libraries Journal	Website	References	no
IEEE Computer	Website	no	no
IEEE Engineering in Medicine and Biology Magazine	Website	References	no
IEEE Pervasive Computing	Website	no	no
IEEE Transactions on Information Technology in Biomedicine	Website	References	no
Informatics in Primary Care	Website	References	no
Informatics Review (The)	Website	no	no
International Journal of Ad Hoc and Ubiquitous Computing	Website	no	no
International Journal of Electronic Healthcare	Website	no	no
International Journal of Healthcare Information Systems and Informatics	Website	no	no
International Journal of Healthcare Technology and Management	Website	no	no
International Journal of Medical Informatics	Website	References	no
International Journal of Technology Assessment in Health Care	Website	References	no
Journal of American Medical Informatics Association (The)	Website	References	Open Access
Journal of Biomedical Informatics	Website	References	no
Journal of Evaluation of Clinical Practice	Website	References	no
Journal of Healthcare Information Management	Website	References	no
Journal of Information Technology in Healthcare	Website	no	no
Journal of Medical Engineering and Technology	Website	References	no
Journal of Medical Internet Research	Website Open Access	References	Open Access
Journal of Medical Systems	Website	References	no
Journal of Telemedicine and Telecare	Website	References	no
Journal of Universal Computer Science	Website Open Access	no	no
Medical and Biological Engineering and Computing	Website	References	no
Medical Decision Making	Website	References	no
Medical Engineering and Physics	Website	References	no
Medical Informatics and the Internet in Medicine	Website	References	no
Methods of Information in Medicine	Website	References	no
Personal and Ubiquitous Computing	Website	no	no
Pervasive and Mobile Computing	Website	no	no
Studies in Health Technology and Informatics	Website	References	no
Technology and Health Care	Website	References	no
Telematics and Informatics	Website	no	no
Telemedicine Journal and e-Health	Website	References	no
Ubiquitous Computing and Communication Journal	Website Open Access	no	no
Ubiquity	Website	no	no

As of November 2007. Related to article “Towards Pervasive Computing in Health Care - A Literature Review”, by Carsten Orwat, Andreas Graefe, Timm Faulwasser
